# Thymidilate synthase and p53 primary tumour expression as predictive factors for advanced colorectal cancer patients

**DOI:** 10.1054/bjoc.1999.0964

**Published:** 2000-01-18

**Authors:** A Paradiso, G Simone, S Petroni, B Leone, C Vallejo, J Lacava, A Romero, M Machiavelli, M De Lena, C J Allegra, P G Johnston

**Affiliations:** Clinical Experimental Oncology Laboratory and Histopathology Service, Medical Oncology, Oncology Institute, via Amendola 209, Bari, 70126, Italy; Grupo Oncologico Cooperativo del Sur, Neuquen, Argentina; Navy Medical Oncology Branch, National Institute of Health, Bethesda, MD, USA; Oncology Department, Queen's University Hospital, Belfast, Northern Ireland, UK

**Keywords:** TS, p53, predictive factor, prognosis, colorectal cancer

## Abstract

The purpose of this work was to analyse the ability of p53 and thymidilate synthase (TS) primary tumour expression to retrospectively predict clinical response to chemotherapy and long-term prognosis in patients with advanced colorectal cancers homogeneously treated by methotrexate (MTX)-modulated–5-fluorouracil (5-FU-FA). A total of 108 advanced colorectal cancer patients entered the present retrospective study. Immunohistochemical p53 (pAb 1801 mAb) and TS (TS106 mAb) expression on formalin-fixed paraffin-embedded primary tumour specimens was related to probability of clinical response to chemotherapy, time to progression and overall survival. p53 was expressed in 53/108 (49%) tumours, while 54/108 (50%) showed TS immunostaining. No relationship was demonstrated between p53 positivity and clinical response to chemotherapy (objective response (OR): 20% vs 23%, in p53+ and p53– cases respectively) or overall survival. Percent of OR was significantly higher in TS-negative with respect to TS-positive tumours (30% vs 15% respectively;*P*< 0.04); simultaneous analysis of TS and p53 indicated 7% OR for p53-positive/TS-positive tumours vs 46% for p53-positive/TS-negative tumours (*P*< 0.03). Logistic regression analysis confirmed a significant association between TS tumour status and clinical response to chemotherapy (hazard ratio (HR): 2.91; 95% confidence interval (CI) 8.34–1.01; two-sided *P*< 0.05). A multivariate analysis of overall survival showed that only a small number of metastatic sites was statistically relevant (HR 1.89; 95% CI 2.85–1.26; two-sided *P*< 0.03). Our study suggests that immunohistochemical expression of p53 and TS could assist the clinician in predicting response of colorectal cancer patients to modulated MTX-5-FU therapy. © 2000 Cancer Research Campaign


*British Journal of Cancer* (2000) **82**(3), 560–567
© 2000 Cancer Research Campaign
DOI: 10.1054/ bjoc.1999.0964, available online at http://www.idealibrary.com on IDEAL®

### Thymidilate synthase and p53 primary tumour expression as predictive factors for advanced colorectal cancer patients

**A Paradiso[1], G Simone[2], S Petroni[2], B Leone[4], C Vallejo[4], J Lacava[4], A Romero[4], M Machiavelli[4], M De Lena[3], CJ Allegra[5] and PG Johnston[6]**

[1]Clinical Experimental Oncology Laboratory and [2]Histopathology Service, [3]Medical Oncology, Oncology Institute, via Amendola 209, 70126 Bari, Italy; [4]Grupo Oncologico Cooperativo del Sur, Neuquen, Argentina; [5]Navy Medical Oncology Branch, National Institute of Health, Bethesda, MD, USA; [6]Oncology Department, Queen's University Hospital, Belfast, Northern Ireland, UK

**Summary** The purpose of this work was to analyse the ability of p53 and thymidilate synthase (TS) primary tumour expression to retrospectively predict clinical response to chemotherapy and long-term prognosis in patients with advanced colorectal cancers homogeneously treated by methotrexate (MTX)-modulated–5-fluorouracil (5-FU-FA). A total of 108 advanced colorectal cancer patients entered the present retrospective study. Immunohistochemical p53 (pAb 1801 mAb) and TS (TS106 mAb) expression on formalin-fixed paraffin-embedded primary tumour specimens was related to probability of clinical response to chemotherapy, time to progression and overall survival. p53 was expressed in 53/108 (49%) tumours, while 54/108 (50%) showed TS immunostaining. No relationship was demonstrated between p53 positivity and clinical response to chemotherapy (objective response (OR): 20% vs 23%, in p53+ and p53– cases respectively) or overall survival. Percent of OR was significantly higher in TS-negative with respect to TS-positive tumours (30% vs 15% respectively; $P < 0.04$); simultaneous analysis of TS and p53 indicated 7% OR for p53-positive/TS-positive tumours vs 46% for p53-positive/TS-negative tumours ($P < 0.03$). Logistic regression analysis confirmed a significant association between TS tumour status and clinical response to chemotherapy (hazard ratio (HR): 2.91; 95% confidence interval (CI) 8.34–1.01; two-sided $P < 0.05$). A multivariate analysis of overall survival showed that only a small number of metastatic sites was statistically relevant (HR 1.89; 95% CI 2.85–1.26; two-sided $P < 0.03$). Our study suggests that immunohistochemical expression of p53 and TS could assist the clinician in predicting response of colorectal cancer patients to modulated MTX-5-FU therapy. © 2000 Cancer Research Campaign

**Keywords**: TS; p53; predictive factor; prognosis; colorectal cancer

Colorectal cancer is the third most common cancer in both men and women in the USA, with about 60 new cases a year per 100 000 inhabitants (SEER Cancer Statistics Review, 1997). Unfortunately, in spite of the advances in the screening and early diagnosis of these patients (Boyle, 1997), 40–45% are destined to metastasize and, as a consequence, to receive systemic chemotherapy. In fact, the combination of 5-fluorouracil (5-FU) and leucovorin (LV) has been shown to improve survival for a significant number of advanced colorectal cancer patients (Machover, 1997). However, the absolute percentage of these patients who will benefit from the chemotherapy is still low and, consequently, indications for this approach warrant further discussion (Isacoff and Borud, 1997). Moreover, due to the lack of predictive factors, the individual response to chemotherapy of advanced colorectal cancer patients is still difficult to foresee. In fact, apart from some clinical factors of limited usefulness (Machiavelli et al, 1991), little is known of the biological and biochemical markers of colorectal tumour responsiveness to fluoropyrimidines.

*Received 21 October 1998*
*Revised 2 February 1999*
*Accepted 16 February 1999*

Correspondence to: A Paradiso. E-mail: anpara@tin.it

With regard to the mechanism of action, 5-FU is effective through its active metabolite, FdUMP, which competes with dUMP for a binding site on the thymidilate synthase (TS) protein, which is the rate-limiting de novo enzyme for synthesis of thymine nucleotides necessary for DNA synthesis. In particular, in vitro evidence suggests that inhibition of TS activity is one of the major mechanisms for the anti-tumoural effect of 5-FU. Spears et al demonstrated that sensitivity to 5FU is partly related to its ability to inhibit TS activity (Spears et al, 1982). In addition, the acute induction of the TS protein and also the stable amplification of TS-specific genes seem to be associated with 5-FU resistance (Johnston et al, 1991). Chu et al suggested that 5-FU resistance is correlated with increased TS levels after being induced by 5-FU with post-transcriptional regulation (Chu et al, 1990). Lastly, Peters et al reported that the LV clinical effect could be related to the prevention of TS induction after 5-FU administration (Peters et al, 1994). All these in vitro studies are concordant in suggesting that 5-FU resistance is effectively related to TS activity in many tumour cell models.

Apart from the above described biochemical mechanisms, it has been recently demonstrated that the cytotoxic effects of 5-FU in some tumour cell lines can be due to apoptosis, the programmed cell death process regulated by p53-dependent mechanisms but which also involves other positive and negative molecular control factors (Merchant et al, 1996). In particular, Lowe et al reported that the p53 wild-type expression is required for 5-FU-induced apoptosis in vitro, and that the acquisition of a p53 mutation renders the tumour cell unresponsive to drugs (Lowe et al, 1994). Furthermore, it has also been demonstrated that in a colorectal cancer cell line bearing an exogeneous wild-type p53 allele, induction of wild-type p53 greatly potentiates 5-FU cytotoxicity (Yang et al, 1996). In fact, a p53 function involved in the determination of clinical response to chemotherapy has been strongly suggested by the demonstration that p53 modulates the activity of the *mdr-1* gene directly implicated in the regulation of a constitutional multidrug cell resistance (Chin et al, 1992). Interestingly enough, in colorectal cancer, a disease generally considered scarcely responsive to systemic therapy, p53 mutations are very common (Smith et al, 1996) and preliminary data indicate that p53 alterations could determine clinical tumour resistance (Bulleco et al, 1996). Recently, stimulating data reported by Lee et al (1997) confirmed that wild-type p53 can directly modulate the expression of TS, which is considered the key enzyme for 5-FU cytotoxic activity.

The above considerations and the recent availability of monoclonal antibodies for TS detection in formalin-fixed tissue sections (Johnston et al, 1991) prompted us to verify if TS and p53 primary tumour expressions evaluated by routine immunohistochemical assays can be of clinical use for predicting clinical response to chemotherapy and long-term prognosis in a large series of homogeneously-treated advanced colorectal cancer patients.

### MATERIALS AND METHODS

### Patient characteristics

A consecutive series of 108 patients with advanced colorectal cancer treated by a first-line 5-FU double modulated chemotherapy in the Institutions of GOCS-Argentina and for whom formalin-fixed paraffin-embedded pathological material was available, were entered in the present retrospective study. A total of 66/108 patients were enroled in CR-01-83 and CR-06-85 clinical trials, while the remaining 42 were consecutively treated after the end of above protocols. Clinical results concerning these protocols have already been published (Machiavelli et al, 1991).

Eligibility criteria for the reported clinical trials included: histological evidence of colorectal cancer without prior chemotherapy for metastatic or advanced recurrent (inoperable) disease, age under 75 years, Eastern Cooperative Oncology Group performance status of 0–3 and a life expectancy of at least 10 weeks.

Immediately after diagnosis, the patients received the following therapeutic schedule: methotrexate (MTX) at 200 mg $m^{-2}$ intravenously (i.v.) by push injection followed after 20 h by 5-FU at 1200 mg $m^{-2}$ administered as continuous i.v. infusion over 2 h. Twenty-four hours after MTX administration all patients received LV at 25 mg i.v. every 6 h for a total of eight doses. Both treatments were repeated every 15 days until disease progression, severe toxicity, or death. To be evaluable for response, patients must have received at least two or more chemotherapy cycles. No radiotherapy was previously or concomitantly administered to any patient.

Standard UICC criteria were used for evaluation of clinical response. Clinical objective response included either complete remission (CR) or partial response (PR) of the disease lasting at least 3 months. The time-to-progression (TTP) was evaluated from the initiation of chemotherapy. Overall survival (OS) in the overall series was computed from start of chemotherapy to death. In accordance with the guidelines proposed by Simes and Zelen (1985), total follow-up time from the onset of treatment for metastatic disease was 103.7 patient years with an average of 11 months (range 1–52) and a failure rate (deaths) of 0.75 failures per year.

Clinical-pathological characteristics of the 108 patients were as follows: median age was 59 years (range 23–74); 74/108 (68%) were males; primary tumour site was colon in 69/108 (24 cases in right colon) and rectum in 39 cases; 49 patients had advanced recurrent disease while 59 had metastatic disease at first diagnosis; lastly, 73 patients had metastatic involvement of the liver. The histopathological grade according to AJCC modified criteria (American Joint Committee on Cancer, 1988) permitted the classification of 26 tumours as well-differentiated (G1), 52 as moderately or poorly-differentiated (G2), and 30 as undifferentiated (G3) tumours.

### Immunohistochemical determination

One formalin-fixed paraffin block of the primary tumour was selected for each patient, based on the quality of the morphologic preservation and neoplastic cellularity. Histological sections of 4 microns were cut and destined to specific immunohistochemical determinations.

The monoclonal antibody (mAb) pAb 1801 used against human p53, reacts with a denaturation-resistent epitope between amino acids 32 and 79 and recognizes both wild-type and mutant forms of the p53 protein.

The mouse monoclonal antibody TS106, kindly provided by PG Johnston (Navy Medical Oncology Branch, NCI, Bethesda, MD, USA) was produced in mice infected with a recombinant human TS protein as already reported (Johnston et al, 1991); this antibody reacts specifically with human TS and displays a negligible cross-reactivity with other cellular proteins of human cells.

To detect masked and unmasked antigen sites both for p53 and TS106, histological sections were deparaffinized and immersed in a 10 mM citrate buffer (pH 6.0) and exposed to microwaves for a 15 min cycle of 750 W using microwaveable slides. The slides were then incubated for 1 h in a humidified atmosphere at room temperature with a 1:400 diluted pAb 1801 monoclonal antibody (Oncogene Science, Uniondale, NY, USA) or a 1:100 diluted TS106 monoclonal antibody to detect the presence of p53 or TS respectively. In the first 20 cases observed, results obtained with the above technique were compared with those using overnight incubation at 4°C with the same dilution of monoclonal antibodies without microwave exposure. We concluded that the sensitivity of the reaction was enhanced in the microwave slides and therefore continued the study utilizing this method.

After incubation, the specimens were washed twice with phosphate-buffered saline (PBS) solution (pH 7.6) and processed with the streptavidin–biotin peroxidase method according to the manufacturer's recommendations. The slides were then incubated in aminoethylcarbazol and hydrogen-peroxide chromogen substrate for 10 min at room temperature, washed in running water for 2–3 min, counterstained in Mayer's haematoxylin and mounted with a permanent medium. The immunoreactivity for p53 antibody was revealed by a dark brown nuclear staining; in sporadic areas of a few tumours, a weak cytoplasmic staining was also observed but not considered in the evaluation of the sample; the immunoreactivity for TS antibody was revealed by a dark brown cytoplasm signal in heterogeneous areas of a few tumours.

© 2000 Cancer Research Campaign

A set of paraffin-embedded colon cancer samples with high levels of p53 and TS expression were used as positive controls while negative controls were obtained by omission of the primary antibody. Tumours cells expressing p53 and TS immunoreactivity were quantified by two independent observers who evaluated at least 1000 neoplastic cells in consecutive areas of neoplastic tissue and numbers of positive cells were then expressed as a percentage of labelled tumour cells with respect to the total number of tumour cells evaluated. Subjective scoring was minimized by regarding all labelled cells as positive irrespective of staining intensity. The sample evaluation was blind in that the investigators had no knowledge of the patient's clinical outcome. Intra- and interlaboratory control of p53 immunohistochemical staining and evaluation has been previously performed (Paradiso et al, 1996).

### Statistical analysis

The p53 and TS status were considered dichotomous variables according to the criteria reported above. Different cut-off values of positive p53 cells from 0% to 50% with a step-wise increase of 5% were utilized for analysis of clinical relationships between p53 primary tumour expression and clinical outcome. For TS analysis, each tumour was classified as positive for any percentage of TS immunostained cells present within the tumour. The cut-off of 0% of TS-positive cells was retained for analysis of prognostic relationships.

The association between p53, TS expression and biological or pathological variables was assessed using the $\chi^2$ test with the Yates correction or Fisher's test. Life-curves were computed by the Kaplan–Meier product-limit method and compared by log-rank test. The joint effect of each presumed prognostic variable was evaluated by a multivariate analysis using the Cox regression model. In this model, the logarithm of the regression coefficients is the hazard ratio which is constant in time. The hazards ratios and their 95% confidence intervals (95% CI) were determined by use of the putative best prognosis category as reference.

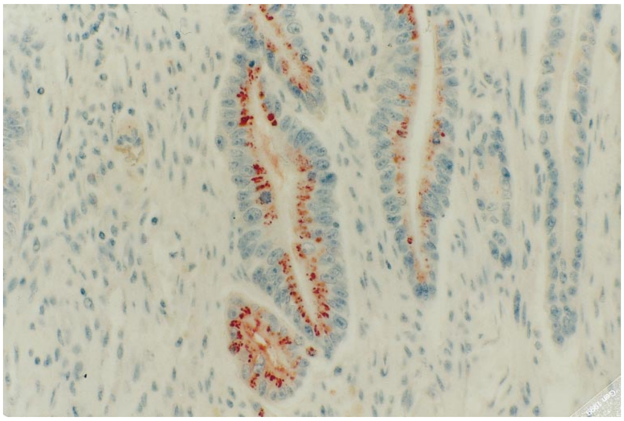

**Figure 1** Immunohistochemical staining of a formalin-fixed paraffin-embedded human colon carcinoma tissue specimens using antibody TS106

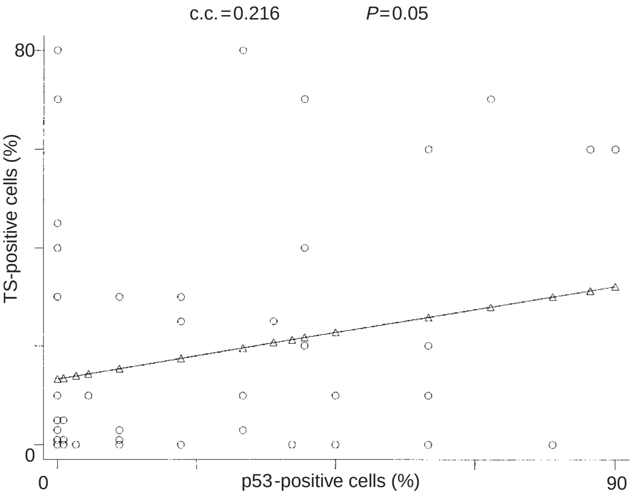

**Figure 2** Percentage of p53-positive cells in relation to percentage of TS-positive cells in the primary tumour of 108 advanced colorectal cancer patients

### RESULTS

Overall, 24/108 patients responded to polychemotherapy. Median length of response was 6 months, whereas median overall survival of the series was 12 months.

p53 expression was evaluable in all 108 cases and was generally confined to neoplastic tissues while normal clonic mucosa was only occasionally stained. Overall, p53 was expressed in 53/108 (49%) tumours with 40 (37%) cases having more than 5% of positive cells. Presence of p53-positive cells (any cut-off value) was not related to sex, primary tumour site (colon vs rectum; left colon vs right colon-rectum), initially advanced or recurrent disease, metastatic site, number of metastases or cytohistological tumour grade.

TS immunoreactivity was generally of the granular type and located at the cytoplasmic level of normal and tumour cells (Figure 1). In normal mucosa the staining intensity was much less than that observed in colorectal tumours and mainly located in the basal area of the cells. All 108 primary tumours were evaluable for TS expression, with 54 (50%) cases showing some kind of immunostaining and 44 (41%) cases with more than 10% of positive tumour cells. Different intensities of TS immunoreactivity were found in cells within the same tumour. TS positivity was significantly higher in females (70% vs 45% respectively; $P < 0.05$) and in G1–2 tumours (68% vs 42% respectively; $P < 0.04$). No association was found between intensity (any cut-off level) and any of the clinical-biological characteristics considered. As previously stated in the Materials and Methods section, the cut-off value of 0% TS-positive cells was retained for further clinical analyses.

When analysing relationships between p53 and TS staining, a strong trend was evident for a direct association between TS and p53 status (positive vs negative) of the individual tumour (overall agreement: 60%; $P > 0.06$); conversely, when percentage of TS- and p53-positive cells in each tumour were considered, a significant but low degree of correlation was shown (c.c. = 0.216; $P < 0.05$) (Figure 2).

With regard to clinical outcome, no statistical association was demonstrated between p53 positivity (cut-off > 5% of positive cells) and probability of clinical response to chemotherapy (objective responses in p53-positive and -negative cases: 20% vs 23% respectively; hazard risk (HR) 1.2; 95% CI 1.3–0.9; $P > 0.5$), length

*© 2000 Cancer Research Campaign*

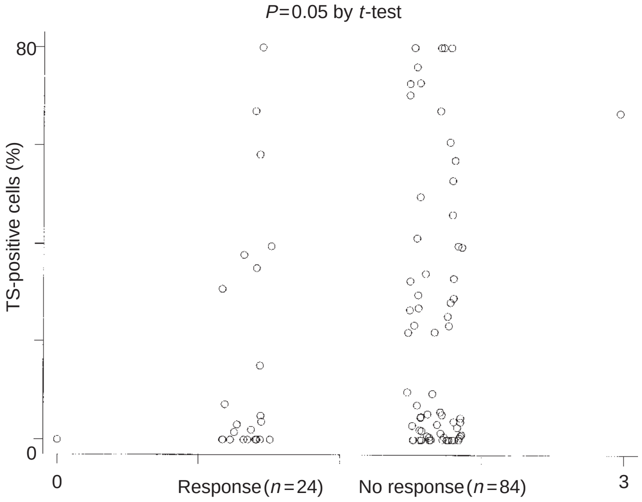

**Figure 3** Distribution of percentage of tumour cells positive for TS immunostaining in a group of 108 advanced colorectal cancer patients with different clinical response to 5-FU-based chemotherapy

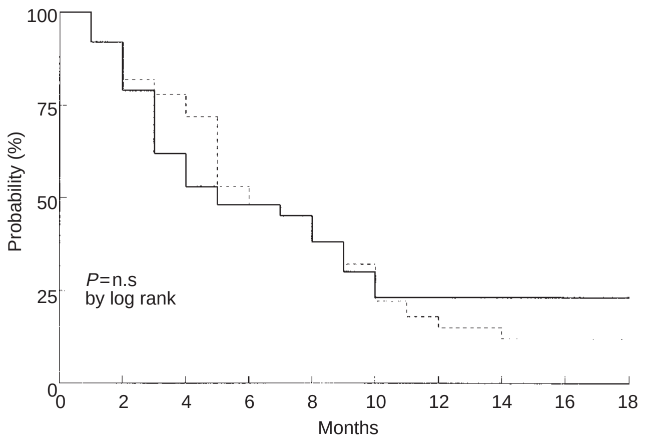

**Figure 4** Time-to-progression (TTP) according to TS status of primary tumour in a series of 108 patients with advanced colorectal cancer (…, TS-negative, $n = 39$; —, TS-positive, $n = 36$)

of response (6 vs 7 months respectively; $P > 0.05$ by log-rank test) and overall survival (11 vs 12 months respectively; $P > 0.05$ by log-rank test). This absence of relationships between p53 status and clinical outcome was also confirmed when different cut-off values for the p53 status were adopted or subsets of patients with different clinical-pathological characteristics were compared (colon vs rectum primary tumour site; recurrent vs initially metastatic disease; liver vs other metastatic sites).

On the contrary, the probability of clinical response to chemotherapy was significantly higher in TS-negative with respect to TS-positive tumours (objective responses: 30% vs 15% respectively; HR 2.0; 95% CI 3.5–1.2; $P < 0.04$). Also the mean TS expression in responder patients was significantly lower than that of non-responders (mean TS expression: 3% vs 31% respectively; $P < 0.05$ by *t*-test) (Figure 3). This association between clinical response and TS negativity was particularly strong in the subset of 69 colon cancer patients (45% vs 15% of clinical responses in TS-negative and -positive colon tumours respectively; $P < 0.02$) compared to the 39 rectal cancer patients (33% vs 26% of clinical responses in TS-negative and -positive rectal tumours respectively; $P$ = n.s.). Furthermore, the median time-to-progression (Figure 4) was the same in TS-negative with respect to TS-positive cases (6 vs 6 months respectively; $P = 0.3$) as was also the overall survival (12 vs 13 months; $P > 0.9$ by log-rank) (Figure 5). This lack of relationship between TS primary tumour expression and long-term clinical outcome was also confirmed when colon and rectal tumours, recurrent and advanced, at first diagnosis were separately considered.

The probability of clinical response to chemotherapy in 108 cases according to TS and p53 status is reported in Table 1. Percentage of clinical response to chemotherapy was 7% for p53-positive/TS-positive tumours vs 46% for p53-positive/TS-negative tumours ($P < 0.03$). Conversely, after stratification according to TS status, p53 did not individualize subgroups of patients with a different percentage of clinical responses. The logistic regression analysis (Table 2) to assess the relative influence of the number of metastatic sites, location of metastatic site, and TS primary tumour expression on the probability of a clinical response to chemotherapy showed that only TS status mantained a significant association with the clinical response of patients to chemotherapy (HR 2.91; 95% CI 8.34–1.01; $P < 0.05$).

Lastly, a Cox multivariate analysis with overall survival as a dependent variable and the same clinical-biological characteristics included in the model showed that only the presence of a lesser

**Table 1** Clinical response to chemotherapy in relation to p53 and TS tumour status in a series of 108 advanced colorectal cancer patients

| Primary tumour TS/p53 status | Clinical response (%) | |
|---|---|---|
| | Response[a] | No response |
| p53-negative/TS-negative | 10 (24%) | 31 (76%) |
| p53-negative/TS-positive | 6 (22%) | 21 (78%) |
| p53-positive/TS-negative | 6 (46%)[b] | 7 (54%) |
| p53-positive/TS-positive | 2 (7%)[b] | 25 (93%) |

Cut-off for p53 positivity: 5% of immunostained cells. Cut-off for TS positivity: ≥ 1% of immunostained cells. [a]Response: complete response or partial response; No response: stable disease or disease progression.
[b]$P < 0.03$ by Fisher's exact test.

**Table 2** Logistic regression analysis of clinical response to chemotherapy in a series of 108 advanced colorectal cancer patients

| Variable | Hazard risk | 95% Confidence interval | *P*-value |
|---|---|---|---|
| TS status[a] (negative vs positive) | 2.91 | 8.34–1.01 | 0.05 |
| No. metastases (1 vs > 1 site) | 1.01 | 1.04–0.98 | 0.5 |
| Site of metastasis (liver vs other) | 1.14 | 1.42–0.81 | 0.8 |

[a] See Table 1 for cut-off value.

© 2000 Cancer Research Campaign

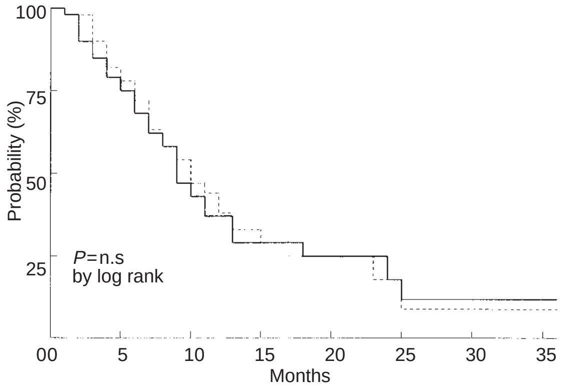

**Figure 5** Overall survival (OS) according to TS status of primary tumour in a series of 108 patients with advanced colorectal cancer (…, TS-negative, *n* = 54; —, TS-positive, *n* = 54)

**Table 3** Cox multivariate analysis of overall survival in a series of 108 advanced colorectal cancer patients

| Variable | Hazard risk | 95% Confidence interval | *P*-value |
|---|---|---|---|
| No. metastases 1 vs > 1 site | 1.89 | 2.85–1.26 | 0.03 |
| TS status negative vs positive[a] | 1.02 | 1.73–0.60 | 0.09 |
| p53 status negative vs positive[a] | 1.12 | 2.02–0.63 | 0.6 |

[a] See Table 1 for cut-off values.

number of metastatic sites maintained an independent association (HR 1.89; 95% CI 2.85–1.26; $P < 0.03$) with overall survival (Table 3).

### DISCUSSION

The p53 tumour suppressor gene, located at chromosomal location 17p13.1, is one of the most commonly altered genes in human solid tumours. Alterations consist in a frequent allelic deletion of the gene and mutations of the remaining allele, generally represented by point mutations located in exons 4–9 (Greenblatt et al, 1994). With respect to the wild-type protein, which has anti-oncogenic characteristics and a short half-life of about 15 min, the mutated p53 protein has a greater stability, a significantly longer half-life and accumulates within malignant cells thus exerting a series of negative effects. Its altered properties include the ability to modify the chemotherapy-induced programmed cell death apoptosis (Lowe et al, 1994); in particular, mutational loss of p53 tumour suppressor functions has been associated with a decreased sensitivity to DNA synthesis blocker drugs such as 5-FU (Mueller and Eppenberger, 1996). Moreover, the possibility to up-regulate the expression of the *mdr-1*, a gene directly involved in pleiotropic multidrug resistance, has been demonstrated for specific p53 mutations (Chin et al, 1992).

Data available on p53 alterations in human colorectal cancers demonstrates an approximate frequency of 50% of point mutations and a percentage of overexpressing tumours varying from 20% to 70% (Smith et al, 1996). Even though controversial results are reported in the literature, the p53 overexpression is thought to be prevalently associated with tumour progression and poor patient prognosis (Greenblatt et al, 1994). On the contrary, studies verifying the capability of p53 status or protein expression to predict the clinical response of colorectal cancer to chemotherapy are scarce, limited to small numbers of patients, and are contradictory. Lenz et al (1998) reported that the p53 status determined by cDNA sequencing was associated with response to 5-FU-based chemotherapy in a series of 36 colorectal cancer patients, while Bulleco et al did not verify any relationship between p53 expression and clinical disease response to hepatic artery infusional fluorouridine in a series of 50 colorectal cancer patients with liver metastases (Bulleco et al, 1996).

In the present study, we update our previous results (Paradiso et al, 1996) analysing the relationships between p53 primary tumour expression and clinical outcome in a larger series of advanced colorectal cancer patients homogeneously treated with a 5-FU-based chemotherapy regimen. We confirm the absence of association between p53 status and clinical response even when different cut-off values for p53 positivity were considered, and patients with colon and rectal cancer with recurrent and initially advanced disease were analysed separately. Contrary to Bulleco et al's results (Bulleco et al, 1996), in our experience also the long-term clinical outcome was not significantly different when plotted against various p53 levels.

Even though comparative studies on advanced colorectal cancer series with similar characteristics are not available, some hypotheses can be forwarded to explain the unexpected absence of a predictive and prognostic relevance for p53 primary tumour overexpression in our study. Several mAbs are now available for the detection of p53 protein reacting at cytoplasmic and nuclear levels (Bosari and Viale, 1994), recognizing different epitopes (Smith et al, 1996) with various degrees of resistance to denaturation (Cook and Milner, 1990); however, their use in clinical series has generated large variations in the p53 characteristics among the different studies. Our negative results could be specific for the mAb pAb 180 which reacts with a denaturation-resistant epitope between amino acids 32 and 79 and recognizes both wild-type and mutant forms of the p53 protein. Consequently, the present results might require further studies in which other mAbs for p53 detection are tested.

Furthermore, the failure to find a significant correlation between p53 primary tumour expression and clinical outcome could be based on the sensitivity and specificity of the immunohistochemical assay for p53 expressions compared to molecular techniques. In fact, strong discrepancies between immunohistochemical and molecular analysis of p53 within the same sample have been reported with a generally higher percentage of p53 alterations resulting with molecular analysis, even if colorectal cancer with p53 accumulation as detected by immunoassays and not harbouring p53 gene mutations has been reported to be quite frequent (Greenblatt et al, 1994). Interestingly enough, this hypothesis has been recently verified by Smith et al in a series of 100 colorectal carcinomas who reported that, while mutations of the p53 gene by molecular assays provides prognostic information, overexpression of the p53 protein as detected by mAbs pAb240 and pAb1801 does not (Smith et al, 1996).

 © 2000 Cancer Research Campaign

It is also possible to hypothesize that the present p53 results, apparently negative, could be interpreted in the light of activity of other genes which positively and negatively control the p53-dependent cell death pathways (Merchant et al, 1996) and also the p53-mediated G1 arrest (Hermeking and Heick, 1994). From this point of view, the role of co-expression of Bcl-2 and p53 oncoproteins (Bathavdekar et al, 1997; Manne et al, 1997), or that of proliferating cell nuclear antigen, p21WAF/Cip1 and p53 expression (Paradiso et al, 1996; Slebos et al, 1996) for prognostic discrimination of colorectal cancer patients have also been recently investigated.

TS is the central enzyme in the metabolic pathway responsible for the final step in the de novo synthesis of thymidilate, a nucleotide required for the synthesis and maintenance of DNA integrity. The expression of the TS gene is believed to be cell cycle associated and high in the S phase (Navalgund et al, 1980), even if preclinical in vitro studies have demonstrated that high intrinsic TS levels do not necessarily mean a higher proliferation rate (Pestalozzi et al, 1995). It has also been suggested that the TS protein regulates the translational efficiency of its own messenger RNA (Chu et al, 1991). In human tissues, high TS levels are generally found in tissues with high cell turnover, such as bone marrow, spleen, thymus and tumour cells (Spears, 1995). Baseline TS levels were found by an enzymatic 3H-FdUMP radioligand binding assay to be higher in primary colon cancers than in liver metastatic sites (Larsson et al, 1996). Moreover, the expression of TS has been verified as an important independent predictor of disease-free survival and overall survival in patients with operable rectal cancer (Johnston et al, 1994) and early breast cancer (Pestalozzi et al, 1997).

In addition to the significance of TS in tumour progression and patient prognosis, considerable effort has been directed to understanding the potential clinical predictive role of this enzyme to determine fluoropyrimidine cytotoxicity. However, available data regard only limited series of colorectal cancer patients generally studied by polymerase chain reaction (PCR) and biochemical techniques. Johnston et al (1995) verified a close association between TS protein and gene expression in response to fluoripyrimidine and folates in nine colorectal cancers and 12 gastric cancer patients. Leichman et al (1997), utilizing a PCR technique to determine TS expression in a series of 46 patients with disseminated colorectal cancer, confirmed the strong association of TS level with clinical response of the tumour to 5-FU, and these data are in agreement with those of Lenz et al in 36 patients (Lenz et al, 1998). Lastly, Peters et al, (1995) and Kornmann et al (1997) confirmed the association of high total tumour TS catalytic activity with clinical resistance of the disease in patients treated with regional chemotherapy.

In these pilot studies, TS quantification was traditionally performed using biochemical or molecular assays, which have some major disadvantages, clearly limiting the possibility for their use in routine clinical activity; the problematic aspects concern the need for large and freshly frozen tumour tissues, the utilization of indirect criteria for molecular quantification of the protein level, the reproducibility of enzymatic assays for evaluation of protein activity, and the need for a well experienced laboratory team. The proof of how relevant is the assay utilized for determination of this enzyme is stressed by the negative-only results reported by Findlay et al (1997) utilizing a polyclonal antibody to recombinant TS and a silver-enhanced immunogold staining with image analysis for TS expression.

Recently, specific mAbs against TS have been synthesized by recombinant techniques and produced in mice (Johnston et al, 1991). Our experience regards the use of clone TS106 of specific TS monoclonal antibodies, which has been shown to be valid for immunological analysis of the protein (Johnston et al, 1991). We performed islet cell antibody assay for TS analysis on paraffin-embedded tumour material for which were available the time of fixation, microwave treatment and time from cutting tumour. Our results are intriguing in that we effectively verified a significant association between positive TS expression and clinical resistance to a fluoropyrimidine-based regimen, demonstrating that TS-positive tumours have only a 15% probability to respond to therapy with respect to 30% for TS-negative tumours ($P < 0.04$). This relationship was also confirmed (HR 2.91; 95% CI 8.34–1.01; $P < 0.05$) with a logistic regression analysis with site and number of metastases included in the model. Therefore, TS seems to be a statistically relevant marker of clinical response to 5-FU-based regimens. However, perhaps because of the relatively low number of patient responders (overall 22% of cases), the median length of response and overall survival did not differ significantly in TS-negative with respect to TS-positive tumours. Johnston et al, analysing in a series of 70 advanced head and neck cancer patients treated with 5-FU-based neoadjuvant chemotherapy, obtained similar results with TS expression relevant in predicting clinical response but not in selecting patients with different long-term clinical outcome (Johnston et al, 1997). On the contrary, Lenz et al utilizing reverse transcription PCR (RT-PCR) assay for TS quantitation in 36 colorectal cancers, reported that TS expression significantly predicts 5-FU tumour response but also a different overall survival (Lenz et al, 1998).

Our last comment concerns the relationship between p53 and TS. By verifying that mean TS levels of tumours with wild-type p53 were significantly lower compared to tumours with mutated p53, Lenz et al hypothesized a regulatory bond between p53 and TS genes (Lenz et al, 1998). Recently, Lee et al confirmed this possibility demonstrating that the wild-type p53 protein is able to inhibit the activity of mouse TS promoter activity (Lee et al, 1997). Our data can be inserted in this scenario as we found different TS characteristics for tumours with different p53 expression; in fact, the percentage of TS-negative tumours was significantly lower in p53-positive than in p53-negative tumours (40% vs 67% respectively; $P < 0.05$). Furthermore, the association between TS status with clinical response significantly differs only in p53-positive (46% vs 8% respectively in TS-negative and -positive tumours) and not in p53-negative tumors (22% vs 24% respectively). In conclusion, taking into account the preliminary data of Lenz et al who obtained the same clinical results utilizing cDNA sequencing for analysis of p53 status and RT-PCR for TS quantitation (Lenz et al, 1998), we can confirm that the role of TS in the modulation of clinical response to fluoropyrimidines seems to be different in tumours with a different p53 status. This is one of the first clinical results directly relating the role of a key enzyme to the cytotoxic effect of a specific drug, such as that of TS for 5-FU, with alterations of p53, a gene able to conduct the tumour cell to apoptotic death and/or G1 arrest.

Our results have been obtained with a relatively easy and feasible technique such as the immunohistochemical assay, which

© 2000 Cancer Research Campaign permits the evaluation of large retrospective series and is of potential routine application in prospective trials. However, we think that further studies on large retrospective series are needed before the application of p53 and TS tumour expression as predictive factors in prospective clinical trials for patients with advanced colorectal cancer.

### ACKNOWLEDGEMENTS

The authors express their appreciation to the following pathologists for submitting the specimens used in the present study: Drs R Scuteri, R Kejner, V Aprea, E Muñoa, G Grossman, G Kremer, A Marchisone, H Matturi, R Ferreyra, O Peralta, C Peralta, F D'Schant, F Marzullo. The authors are also grateful to Ms I Trotti for technical collaboration and to Ms MP Butts for her assistance in the preparation of the manuscript. Elaboration of data have been performed by Baldassarre Stea. The authors wish to thank Prof. G Viale (European Institute of Oncology, Milan) for interlaboratory quality control of p53 immunohistochemical analysis. Project partially supported by AIRC, CNR, and Italian Ministry of Health; Project included in Pluriennal Bilateral Italy–Argentina Cooperative Program.

### REFERENCES

American Joint Committee on Cancer (1988) *Manual for Staging of Cancer* 3rd edn, p.7. Lippincott: Philadelphia

Bathavdekar JM, Patel DD, Ghosh N, Chikhlikar PR, Trivedi TI, Suthar TP, et al (1997) Coexpression of Bcl-2, c-myc, and p53 oncoproteins as prognostic discriminants in patients with colorectal carcinoma. *Dis Colon Rectum* **40:** 785–790

Bosari S and Viale G (1994) Cytoplasmic accumulation of p53 protein: an independent prognostic indicator in colorectal adenocarcinomas. *J Natl Cancer Inst* **86:** 681–687

Boyle P (1997) Global burden of cancer. *Lancet* **349:** 23–26

Bulleco C, Guillem JG, Kemeney N, Huang Y, Klimistra D, Berger MF, et al (1996) p53 nuclear protein overexpression in colorectal cancer: a dominant predictor of survival in patients with advanced hepatic metastases. *J Clin Oncol* **14**: 2696–2701

Chin KV, Ueda K, Pastran I and Gottesman MM (1992) Modulation of activity of the promoter of the human MDR1 gene by RAS and p53. *Science* **255**: 459–462

Chu E, Drake JC, Koeller DM, Zinn S, Jamis-Dow CA, Yeh GC, et al (1990) Induction of thymidilate synthase associated with multidrug resistance in human breast and colon cancer cell lines. *Mol Pharmacol* **39**: 136–143

Chu E, Koeller DM, Casey JL, Drake JC, Chabner BA, Elwood BC, et al (1991) Autoregulation of human thymidilate messenger RNA translation by thymidilate synthase. *Proc Natl Acad Sci USA* **88**: 8977–8981

Cook A and Milner J (1990) Evidence of allosteric variants for wild type p53, a tumour suppressor protein. *Br J Cancer* **61**: 548–562

Edler D, Blomgren H, Allegra CJ, Johnston PG, Lagerstedt U, Magnusson I and Ragnhammar P (1997) Immunohistochemical determination of thymidylate synthase in colorectal-cancer-methodological studies. *Eur. J. Cancer* **33**: 2278–2282

Findlay MPN, Cunningham D, Morgan G and Clinton S (1997) Lack of correlation between thymidylate synthase levels in primary colorectal tumors and subsequent response to chemotherapy. *Br J Cancer* **75**: 903–909

Greenblatt MS, Bennett WP, Hollstein M and Harris CC (1994) Mutations in the p53 tumor suppressor gene: clues to cancer etiology and molecular pathogenesis. *Cancer Res* **54**: 4855–4878

Hermeking H and Heick D (1994) Mediation of c-myc-induced apoptosis by p53. *Science* **265**: 2091–2093

Isacoff WH and Borud K (1997) Chemotherapy for the treatment of patients with metastatic colorectal cancer: an overview. *World J Surg* **21**: 748–762

Johnston PG, Liang CM, Henry S, Chabner BA and Allegra C (1991) Production and characterization of monoclonal antibodies that localize human thymidilate synthase in the cytoplasm of human cells and tissue. *Cancer Res* **51**: 6668–6676

Johnston PG, Fisher ER, Rockette HE, Fisher B, Wolmark N, Drake JC, et al (1994) The role of thymidilate synthase expression in prognosis and outcome of adjuvant chemotherapy in patients with rectal cancer. *J Clin Oncol* **12**: 2640–2647

Johnston PG, Lenz HJ, Leichman CG, Danenberg K, Allegra C, Danenberg PV, et al (1995) Thymidilate synthase gene and protein expression correlate and are associated with response to 5-Fluorouracil in human colorectal and gastric cancer. *Cancer Res* **55**: 1407–1412

Johnston PG, Mick R, Recant W, Behan KA, Dolan ME, Ratain MJ, et al (1997) Thymidilate synthase expression and response to neoadjuvant chemotherapy in patients with advanced head and neck cancer. *J Natl Cancer Inst* **89**: 308–313

Kornmann M, Link KH, Lenz HJ, Pillasch J, Metzger R, Butzer U, et al (1997) Thymidylate synthase is a predictor for response and resistance in hepatic artery infusion. *Cancer Letter* **118**: 29–35

Larsson PA, Carlsson G, Gustavsson B and Spears PC (1996) Thymidilate synthase in advanced gastrointestinal and breast cancers. *Acta Oncologica* **35**: 469–472

Lee Y, Chen Y, Chang LS and Johnson LF (1997) Inhibition of mouse thymidilate synthase promoter activity by the wild-type p53 tumor suppressor protein. *Exp Cell Res* **234**: 270–276

Leichman CG, Lenz HJ, Leichman L, Danenberg K, Baranda J, Groshen S, et al (1997) Quantitation of intratumoral thymidilate synthase expression predicts for disseminated colorectal cancer response and resistance to protracted-infusion fluorouracil and weekly leucovorin. *J Clin Oncol* **15**: 3223–3229

Lenz HJ, Hayashi K, Salonga D, Danenberg KD, Danenberg PV, Metzger T, Banerijee D, Bertino JR, Groshen S, Leichman LP and Leichman CG (1998) p53 point mutations and Thymidylate synthase messenger RNA levels in disseminated colorectal cancer: an analysis of response and survival. *Clin. Cancer Res.* **4**: 1243–1250

Lowe SW, Bodis S, McClatchey A, Remington L, Ruley HE, Fisher DE, et al (1994) p53 status and the efficacy of cancer therapy in vivo. *Science* **266**: 807–810

Machiavelli M, Leone BA, Romero A, Rabinovich MG, Vallejo CT, Bianco A, et al (1991) Advanced colorectal carcinoma. *Am J Clin Oncol* **14**: 211–217

Machover D (1997) A comprehensive review of 5-fluorouracil and leucovorin in patients with metastatic colorectal carcinoma. *Cancer* **80**: 1179–1187

Manne U, Myers RB, Moron C, Poczatek RB, Dillard S, Weiss H, et al (1997) Prognostic significance of Bcl-2 expression and p53 nuclear accumulation in colorectal adenocarcinoma. *Int J Cancer* **74**: 346–358

Merchant AK, Loney TL and Maybaum J (1996) Expression of wild-type p53 stimulates an increase in both Bax and Bcl-x(L) protein content in HT29 cells. *Oncogene* **13**: 2631–2637

Mueller H and Eppenberger U (1996) The dual role of mutant p53 protein in chemosensitivity of human cancer. *Anticancer Res* **16**: 3845–3848

Navalgund LG, Rossann C, Muench AJ and Johnson LF (1980) Cell cycle regulation of thymidilate synthase gene expression in culture mouse fibroblasts. *J Biol Chem* **255**: 7386–7390

Paradiso A, Rabinovich M, Vallejo C, Machiavelli M, Romero A, Perez J, et al (1996) p53 and PCNA expression in advanced colorectal cancer. Response to chemotherapy and long-term prognosis. *Int J Cancer* **69**: 437–441

Pestalozzi BC, McGinn CJ, Kinsella TJ, Drake JC, Glennon MC, Allegra CJ, et al (1995) Increased thymidilate synthase protein levels are principally associated with proliferation but not cell cycle phase in asynchronous human cancer cells. *Br J Cancer* **71**: 1151–1157

Pestalozzi BC, Peterson HF, Gelber RD, Goldhirsch A, Gusterson BA, Trihia H, et al (1997) Prognostic importance of thymidilate synthase expression in early breast cancer. *J Clin Oncol* **15**: 1923–1931

Peters GJ, Van Der Wilt CL, Van Groeningen CJ, Meijer S, Smid K and Pinedo HM (1994) Thymidilate synthase inhibition after administration of 5-fluorouracil with or without leucovorin; implications for treatment with 5-fluorouracil. *J Clin Oncol* **12**: 2035–2042

Peters GJ, Van Der Wilt CL, Van Triest B, Codacci-Pisanelli G, Johnston PG, Van Groeningen CJ, et al (1995) Thymidylate synthase and drug resistance. *Eur J Cancer* **31(7)**: 1299–1305

SEER Cancer Statistics Review, 1973–1993, preliminary edition (1997) Colorectal cancer incidence. *J Natl. Cancer Inst* **89**: 416

Simes RJ and Zelen M (1985) Exploratory data analysis and the use of the hazard function for interpreting survival data: an investigator's prime. *J Clin Oncol* **3**: 1418–1431

 *© 2000 Cancer Research Campaign*

Slebos RJC, Baas IO, Clement M, Polak M, Mulder JW, Van Den Berg FM, et al (1996) Clinical and pathological associations with p53 tumor-suppressor gene mutations and expression of p21WAF1/Cip1 in colorectal carcinoma. *Br J Cancer* **74**: 165–171

Smith DR, Ji CY and Goh HS (1996) Prognostic significance of p53 overexpression and mutation in colorectal adenocarcinomas. *Br J Cancer* **74**: 216–223

Spears C (1995) Clinical resistance to antimetabolites. *Haematol Oncol Clin North Am* **9**: 397–413

Spears CP, Shahanian AH and Moran RG (1982) In vivo kinetics of thymidilate synthase inhibition of 5-fluorouracil-sensitive and resistant murine colon adenocacarcinomas. *Cancer Res* **42**: 450–456

Yang B, Eshleman JR, Berger NA and Markowitz SD (1996) Wild-type p53 potentiates cytotoxicity of therapeutic agents in human colon cancer cells. *Clin Cancer Res* **2**: 1649–1657

© 2000 Cancer Research Campaign
